# Chronic fatigue: psychometric properties and updated norm values of the Chalder fatigue scale in a cross-sectional sample representative of the German population

**DOI:** 10.1080/07853890.2025.2524087

**Published:** 2025-07-11

**Authors:** Lina Krakau, Felix Wicke, Winfried Häuser, Manfred E. Beutel, Elmar Brähler, Nora Hettich-Damm

**Affiliations:** a Department for Psychosomatic Medicine and Psychotherapy, University Medical Center of the Johannes Gutenberg University Mainz, Mainz, Germany; b Department Psychosomatic Medicine and Psychotherapy, Technical University Munich, München, Germany; c Integrated Research and Treatment Center Adiposity Diseases, Behavioral Medicine Unit, Department of Psychosomatic Medicine and Psychotherapy, Leipzig University Medical Center, Leipzig, Germany

**Keywords:** Chronic fatigue, fatigue, measurement, somatic symptoms, Chalder Fatigue Scale

## Abstract

**Background:**

The Chalder Fatigue Scale (CFQ) is a commonly used self-report instrument assessing the severity and chronicity of fatigue. We examine the psychometric properties of the CFQ and provide updated normative data for the German general population.

**Materials and Methods:**

The CFQ was administered to *N* = 2519 participants (16-96 years). Statistical analyses included the evaluation of the item properties, confirmatory factor analysis and examinig associations with mental health and sociodemographic data. CFQ cut-offs were used to estimate proportions of severe and chronic fatigue scores. We calculated percentile norms for the total sample and stratified by age groups and gender.

**Results:**

Indicators of internal consistency reliability were high for the CFQ total and subscales (*α* = 0.84–0.94; *ω* = 0.86–0.95). We found excellent model fit for a one (*χ*^2^ = 196.011, *df* = 44, *p* ≤ .001; CFI=.991, RMSEA = 0.037, SRMR = 0.059) and two-factor solution (*χ*^2^ = 115.055, *df* = 42, *p* ≤ .001; CFI=.996, RMSEA = 0.026, SRMR = 0.045). The CFQ total scale showed low to moderate associations with depression (*r* = .49, *p* ≤ .001), anxiety (*r* = .45, *p* ≤ .001), and loneliness (*r* = .26, *p* ≤ .001), indicating acceptable discriminant validity. Current unemployment was a relevant sociodemographic correlate of fatigue severity (CFQ total: *β* =.38, se =.09, *p* ≤ .001). 14.2% of participants reported severe fatigue, while 4.3% reported being fatigued for at least six months (chronic fatigue).

**Conclusion:**

The CFQ is a brief and reliable instrument for assessing fatigue in general population settings. The results are limited by the lack of comparison with other established fatigue questionnaires.

## 1. Introduction

Fatigue is a significant and often debilitating symptom in several medical conditions such as cancer, neurological disorders such as multiple sclerosis, infectious diseases, somatic symptom disorders, anxiety, and depression [1,2], and it has also become a leading symptom of post/long-COVID-19 [3]. It is the primary or secondary reason for 10%–20% of primary care consultations [4], but the multitude of associated or underlying medical conditions is recognized as complicating the diagnostic process for clinicians [5]. Mild fatigue is commonly experienced in the general population [6], but severe fatigue that is not alleviated by normal regeneration interferes with daily functioning and is a significant burden. This highlights the need to continually update normative data on measures of fatigue and chronic fatigue (CF), and to monitor prevalence in population-based samples.

Physical (e.g. reduced strength), cognitive (e.g. lack of concentration), and emotional (e.g. lack of energy) fatigue symptoms may be distinguished [4]. The persistence of symptoms for at least six months is considered chronic fatigue (CF). Different criteria and case definitions have been proposed for the diagnosis of chronic fatigue syndrome/myalgic encephalomyelitis (CFS/ME) or systemic exertion intolerance disease (SEID), the term recommended by the Institutes of Medicine [7]. Based on different definitions and assessment methods, prevalence estimates of 4 to 45% have been reported for severe fatigue in general population samples [2,8] and 2%–11% [8,9] for CF. Much lower prevalence rates of 0.007%–2.8% have been estimated for the diagnosis of CFS/ME [9–12].

The Chalder Fatigue Scale (CFQ, 13] is a commonly used multidimensional instrument for assessing the severity and chronicity of fatigue. It is suitable for evaluating change following interventions [14]. The scale has been successfully applied in different cultural contexts [15–20] and to study fatigue associated with different conditions [14,18,21]. The 11-item CFQ is a brief screening instrument that can be used for epidemiological and clinical purposes. The abbreviation CFQ is commonly used to distinguish it from CFS. The scoring procedure of the instrument allows to differentiate between no fatigue and severe fatigue with an additional item to assess chronicity. According to Cella & Chalder [22], the scale accurately discriminates between CFS cases and non-cases, especially at higher scores. Compared to other instruments, it may capture a wider range of fatigue severity, suggesting its usefulness in population-based and research settings [19]. The CFQ is designed to capture physical (e.g. tiredness) and mental (e.g. impaired memory) dimensions of fatigue. Debates about the dimensionality of fatigue are also reflected in various uni- and multidimensional questionnaires to assess fatigue and its components [23,24]. Martin et al. [20] found support for the two-factor solution of the CFQ in the German general population. Based on data from 2004, they found that 7.4% of participants reported severe fatigue, and 6.1% reported CF. In the present study, we aimed to replicate the findings of Martin et al. [20] on the psychometric properties of the instrument and to provide updated normative data for the CFQ total scale and both subdimensions, physical and mental fatigue.

## 2. Methods

### 2.1. Study design

The study was approved by the ethics committee of the University of Leipzig (474/20-ek). Data collection was carried out with the assistance of the German consulting firm USUMA. Target households were randomly selected from 258 sample points in Germany using a random route procedure. Kish-selection [25] was used to identify target individuals within households to ensure a randomly selected sample representative of the German general population. Participants were required to be at least 16 years of age, to have sufficient understanding of the German language, and consent to participate in the study. No other inclusion or exclusion criteria were applied. After giving verbal informed consent, participants were interviewed at their homes. According to the German Federal Data Protection Act (Bundesdatenschutzgesetz §4a) verbal consent is sufficient for fully anonymized social and marketing research, which is not affected by the European Union General Data Protection Regulation. Trained interviewers collected socio-demographic data, and participants completed self-report questionnaires on mental health, health behavior, and attitudes. Data were collected between December 2020 and February 2021. *N* = 2519 participants were recruited and completed the survey, including 1,322 (52.5%) women, 1193 (47.4%) men and 4 (0.2%) gender-diverse individuals with a mean age of 50.33 years (min: 16, max: 96). The response rate was 42.6%. The authors affirm that all procedures contributing to this work complied with the ethical standards of the relevant national and institutional committees on human experimentation and with the Helsinki Declaration of 1975, as revised in 2008.

### 2.2. Measures

#### 2.2.1. Fatigue

We assessed participant’s fatigue using the German version [20] of the Chalder Fatigue Scale [13]. The CFQ consists of a global scale and two subscales, physical and mental fatigue. On eleven items, participants rate their fatigue over the past month compared to the last time they felt well. The anchors of item 1–10 are: less (0), no more (1), more (2) or much more (3) than usual. Item 11 reads: better (0) no worse (1) worse (2) much worse (3) than usual. The sum of all items (0–33) represents the global fatigue severity. The two sub-dimensions represent the sum of items 1–7 (0–21) for physical fatigue, and 8–11 (0–12) for mental fatigue. To identify cases with severe fatigue levels each item value can be transformed into a binary score (0 and 1 = 0; 2 and 3 = 1). When calculating the total sum score of the binary scoring procedure, a total score of 4 or more indicates severe fatigue. Chronicity is assessed with an additional item indicating the persistence of symptoms for at least six months.

#### 2.2.2. Mental distress

The PHQ-4 [26,27] is a screening measure for general mental distress. Participants rate four items on a 4-point Likert scale (0 = not at all to 3 = nearly every day) related to the frequency of depressed mood or worry over the two weeks. In addition to the total distress score, sum scores for depression (PHQ-2) or general anxiety (GAD-2) can be calculated. Cut-offs of 6 or greater for the total scale and 3 or greater for each subscale have been recommended as cut-offs for clinical population. Acceptable to good internal consistency has been found in German general population with *ω*=.85 for the total scale, *ω*=.78 for the depression and *ω*=.77 for the anxiety subscales [28].

#### 2.2.3. Loneliness

Loneliness was measured using a validated single-item screening instrument [29]. Participants were asked whether they were ‘frequently alone/have few contacts’ and rated the answer on a 5-point Likert scale (0 = no, does not apply, 1 = yes, it applies, but I do not suffer from it, 2 = yes, it applies, and I suffer slightly, 3 = yes, it applies, and I suffer moderately, 4 = yes, it applies, and I suffer strongly).

### 2.3. Statistical analyses

All analyses were performed in R version 4.3.0 [30]. We set statistical significance for all tests at *p* < 0.05 (two-tailed). Effect sizes are interpreted according to Cohen [31,32], where *d* = 0.2 and *r* = 0.10 are considered small, *d* = 0.5 and *r* = 0.30 medium, and *d* = 0.8 and *r* = 0.50 large effects. We report Cronbach’s alpha and McDonald’s omega as measures of reliability. Non-response rates were calculated for each item and were expected to be less than 5%. We used listwise deletion to handle missing data. We considered floor and ceiling effects when more than 15% of participants reported the lowest or highest possible score, respectively. Deviations from the normal distribution were assessed using skew and kurtosis and the Kolmogorov-Smirnov test. We assessed the factor structure of the CFQ using confirmatory factor analysis (CFA). We compared a one-factor solution including all items with a two-factor solution. The two-factor solution was modeled according to the proposed subdimensions of the CFQ, including physical fatigue (item 1–7) and mental fatigue (item 8–11). We used a robust weighted least squared approach, namely, the WLSMV estimator of the R-package lavaan [33], which handles non-normal distributions in items with few categories [34]. We used a set of standard goodness of fit statistics to evaluate model fit. According to West et al. [35], we accepted models with a Standardized Root Mean Square-Residual (SRMR) <.08, a Root Mean-Square Error of Approximation (RMSEA) <.06 and a Comparative Fit Index (CFI) >.95. Factor loadings ≥ 40 were considered to contribute meaningfully to the scale. We analyzed the discriminant validity of the CFQ total score and both subscales (physical and mental fatigue) using Pearson correlations with the single-item loneliness measure, the PHQ-2 (depression), the GAD-2 (anxiety), the PHQ-4 (mental distress). We also compared fatigue severity between participants with and without clinically relevant levels of depression and anxiety on all CFQ scales using Wilcoxon rank-sum test. We performed multiple linear regression analysis to determine associations with sociodemographic and socioeconomic variables and fatigue severity. The variables examined included: age, gender (men/women), immigrant background (yes/no), education level (high school graduate/no high school graduate), current unemployment (yes/no), religious affiliation (yes/no), living in a metropolitan area, living in a steady partnership (yes/no), living with children (yes/no), living alone (yes/no). We calculated percentile norms of the CTQ total scale and both subdimensions (physical and mental fatigue) for the total sample and stratified by age groups and gender. The percentile rank indicates how many percent of the sample population report a score equal to or lower than the given score. Gender normative data could only be provided for individuals who identified as men or women, as only four participants reported diverse gender. Frequency estimates for severe fatigue and chronicity were calculated according to the CFQ cut-offs as described in Section 2.2.

## 3. Results

An overview of the sample is reported in Table 1.

**Table 1.** Sample description.

		Total sample
		*N* = 2519
Gender *N* (%)	Diverse	4 (0.2)
	Women	1322 (52.5)
	Men	1193 (47.4)
Age *M*(SD)		50.33 (18.06)
Partnership *N* (%)	No	1011 (40.8)
	Yes	1468 (59.2)
High school graduate *N* (%)	No	1011 (73.9)
	Yes	652 (26.1)
Currently unemployed *N* (%)	No	2367 (94.0)
	Yes	152 (6.0)
Equivalized income *M*(SD)		1972.85 (960.56)
Religiously affiliated *N* (%)	No	769 (30.9)
	Yes	1720 (69.1)
Living with child(ren) under 16 years *N* (%)	No	2081 (82.6)
	Yes	438 (17.4)
Migration background *N* (%)	No	2208 (87.7)
	Yes	311 (12.3)
Living alone *N* (%)	No	1613 (64.0)
	Yes	906 (36.0)
Living in a metropolitan area *N* (%)	No	574 (22.8)
	Yes	1945 (77.2)

*Note*. SD: standard deviation, *population ≥ 100,000.

### 3.1. Descriptive analyses and psychometric properties

Table 2 displays characteristics of all items and the CFQ total and subscales. We found a low rate of missingness, ranging between 0.04% and 0.28% on all items, resulting in 1.15% on the total scale. Mean item scores vary between 0.842 (SD = 0.512; *Difficulties Thinking Clearly*) and 1.038 (*SD* = 0.626; *Lack of Energy*). Positive skewness was found for the total scale (.36) and both subscales (physical =.52; mental=.30) indicating that the distribution was right skewed. The kurtosis of the total (5.19) and both subscales (physical = 4.97; mental = 5.60) indicated a more heavy-tailed distribution compared to the normal distribution. Mean total severity was 10.59 (SD = 4.81) with 6.93 (*SD* = 3.52) for the physical and 3.67 (*SD* = 1.66) for the mental subscale. The median was of the total scale was 11 (CFQ_Physical_ = 7, CFQ_Mental_ = 4). Based on the Kolmogorov Smirnov Test, we found significant deviations from the normal distribution on all scales (CFQ_Total_: *D* = 0.68, *p* <.001; CFQ_Physical_: *D* = 0.68, *p* <.001; CFQ_Mental_: *D* = 0.68, *p* <.001). Participants endorsed the full range of possible index values, with a minimum of 0 and maximums of 33, 21, and 12, respectively. A floor effect is indicated by the fact that approximately 15% or more of the participants scored zero on 10 out of the 11 fatigue items. Table 3 shows the psychometric properties of the items and the item intercorrelations. All items are moderately to highly correlated with each other. The part-whole corrected item-total correlation ranged from 0.52 to 0.81. We found good internal consistency for the total score (*α* = 0.94, *ω* = 0.95) and for the physical (*α* = 0.94, *ω* = 0.95) and mental (*α* = 0.84, *ω* = 0.86) subscales. Guttman’s coefficient for test split-half reliability was also high (λ6_total_ = 0.95, λ6_physical_ = 0.93, λ6_mental_ = 0.81). Dropping one item resulted in Cronbach’s alpha coefficients between 0.93 and 0.94, and McDonald’s omega coefficients between 0.95 and 0.96. Overall, the scale shows good psychometric properties.

**Table 2.** Descriptive item and scale characteristics of the Chalder Fatigue Scale (CFQ).

						Item Score *N* (%)			
CFQ Item	*N* valid	MV *N* (%)	*M*	*SD*		0	1	2	3
1.Do you have problems with tiredness?	2518	1 (0.04)	0.994	0.570		371 (14.7)	1835 (72.9)	267 (10.6)	45 (1.8)
2.Do you need to rest more?	2518	1 (0.04)	1.002	0.572		367 (14.6)	1820 (72.3)	289 (11.5)	42 (1.7)
3.Do you feel sleepy or drowsy?	2512	7 (0.28)	1.007	0.581		370 (14.7)	1800 (71.7)	297 (11.8)	45 (1.8)
4.Do you have problems starting things?	2518	1 (0.04)	0.994	0.626		445 (17.7)	1699 (67.5)	318 (12.6)	56 (2.2)
5.Do you lack energy?	2517	2 (0.08)	1.038	0.626		393 (15.6)	1690 (67.1)	380 (15.1)	54 (2.1)
6.Do you have less strength in your muscles?	2516	3 (0.11)	0.970	0.602		439 (17.4)	1768 (70.3)	254 (10.1)	55 (2.2)
7.Do you feel weak?	2515	4 (0.16)	0.927	0.593		501 (19.9)	1,735 (69.0)	240 (9.5)	39 (1.6)
8.Do you have difficulties concentrating?	2517	2 (0.08)	0.957	0.582		441 (17.5)	1784 (70.9)	251 (10)	41 (1.6)
9.Do you make slips of the tongue when speaking?	2517	2 (0.08)	0.863	0.512		506 (20.1)	1871 (74.3)	120 (4.8)	20 (0,8)
10.Do you find it more difficult to find the correct word?	2512	7 (0.28)	0.842	0.512		546 (21.7)	1832 (72.9)	120 (4.8)	14 (0.6)
11.How is your memory?	2509	10 (0.40)	1.002	0.391		179 (7.1)	2155 (85.9)	165 (0.6)	10 (0.4)
Sum Score	*N* valid	MV *N* (%)	*M*	*SD*	*MD*	*Min*	*Max*	*Skew*	*Kurtosis*
CFQ Total	2490	29 (1.15)	10.594	4.891	11	0	33	0.361	5.190
CFQ Physical	2506	13 (0.52)	6.932	3.524	7	0	21	0.524	4.970
CFQ Mental	2502	17 (0.67)	3.665	1.656	4	0	12	0.299	5.601

Note. M: mean, MV: missing values, SD: standard deviation.

**Table 3.** Item characteristics of the CFQ.

				Item inter correlations (*r*II)										
CFQ Item	α – I	ω-I	*r*IT	1	2	3	4	5	6	7	8	9	10	11
1	0.93	0.95	0.76	1										
2	0.93	0.95	0.76	0.76	1									
3	0.93	0.95	0.80	0.78	0.77	1								
4	0.93	0.95	0.78	0.62	0.61	0.65	1							
5	0.93	0.95	0.81	0.67	0.65	0.70	0.78	1						
6	0.93	0.95	0.73	0.57	0.61	0.60	0.60	0.64	1					
7	0.93	0.95	0.81	0.61	0.65	0.67	0.67	0.68	0.74	1				
8	0.93	0.95	0.79	0.58	0.57	0.63	0.68	0.68	0.59	0.69	1			
9	0.94	0.95	0.66	0.46	0.46	0.49	0.53	0.53	0.52	0.57	0.63	1		
10	0.94	0.95	0.70	0.48	0.48	0.52	0.57	0.56	0.52	0.61	0.68	0.75	1	
11	0.94	0.96	0.52	0.41	0.41	0.42	0.41	0.42	0.39	0.43	0.45	0.41	0.47	1

*Note*. α – I: Cronbach’s alpha of the CFQ-sum when the respective item is deleted. ω-I: Mc Donald’s omega of the CFQ-sum when the respective item is deleted. rIT: part whole corrected item-total correlation.

### 3.2. Factor structure

We fit a one-factor CFA, in which all items loaded on a global factor, and compared it with a two-factor CFA, which was fit according to the postulated structure of the CFQ (items 1–7 loading on a latent physical fatigue factor, items 8–11 loading on a latent mental fatigue factor). The one-factor solution showed an excellent model fit (*χ*^2^ = 196.011, *df* = 44, *p* < 0.001; CFI=.991, TLI=.989, RMSEA = 0.037, SRMR = 0.059) with factor loadings ranging from .53 to .84. The two-factor solution showed only a slightly better fit (*χ*^2^ = 115.055, *df* = 42, *p* < 0.001; CFI=.996, TLI=.995, RMSEA = 0.026, SRMR = 0.045), with factor loadings ranging from .77 to .85 for the physical subfactor and from .58 to .89 on the mental subfactor. Physical and mental fatigue were highly correlated (.84). By standard criteria both models showed excellent fit, with the one-factor solution being the more parsimonious model.

### 3.3. Discriminant validity

Table 4 shows the intercorrelations of the CFQ total score, the subscales, and individual items with loneliness, depressive symptoms (PHQ-2), anxiety symptoms (GAD-2), and the PHQ-4 total score. We found moderate correlations between the CFQ and the PHQ-4 scales. Low correlations were found between loneliness and the CFQ scales. The correlation of loneliness with the CFQ items was low, and low to moderate correlations were found for the PHQ scales with the CFQ items. *N* = 184 participants scored ≥ 3 on the PHQ-2, indicating clinically relevant levels of depressive symptoms. For anxiety (GAD-2), this was true for *N* = 157 participants. We performed Wilcoxon rank sum tests, to compare CFQ scale scores of participants with and without clinically relevant symptom levels of depression and anxiety (cut-offs of the respective questionnaires). The mean CFQ total score for participants with clinically relevant depression symptoms was statistically significantly higher (*M* = 17.27, SD = 6.47, *Mdn* = 17) compared to those without (*M* = 10.05, SD = 4.31, *Mdn* = 11), with a medium effect size (*W* = 70,604, *p* < 0.001, *r* = 0.32). The picture was similar for both subscales. Participants with clinically relevant levels of depression scored higher (CFQ_physical_ *M* = 11.83, SD = 4.61, Mdn = 12; CFQ_mental_ *M* = 5.42, *SD* = 2.30, Mdn = 5) than those without (CFQ_physical_ *M* = 6.54 *SD* = 3.10, Mdn = 7; CFQ_mental_ *M* = 3.52, SD = 1.51, Mdn = 4). The differences were statistically significant and slightly larger for the CFQ physical subscale (W_physical_ = 70,894, *p* < 0.001, *r* = 0.32; W_mental_ = 97,026, *p* < 0.001, *r* = 0.28). Similarly, participants with clinically relevant levels of anxiety scored statistically significantly higher on the CFQ total (*M* = 17.21, *SD* = 6.86, Mdn = 17 vs. *M* = 10.15, SD = 4.39, Mdn = 11, *W* = 64,897, *p* < 0.001, *r* = 0.28), the CFQ physical (*M* = 11.60, SD = 4.81, Mdn = 12 vs. *M* = 6.62, *SD* = 3.18, Mdn = 7, *W* = 68,172, *p* < 0.001, *r* = 0.28), and the CFQ mental scale (*M* = 5.57, *SD* = 2.44, Mdn = 4 vs. *M* = 3.54, SD = 1.51, Mdn = 5, *W* = 83,990, *p* < 0.001, *r* = 0.26). In summary, the low to moderate correlations of the CFQ scales with indicators of mental distress, and the elevated levels of fatigue in populations reaching clinical cut-offs of depression and anxiety indicate acceptable discriminant validity of the CFQ.

**Table 4.** Correlations of fatigue with loneliness, depression, and anxiety.

	Loneliness (GHS-Item)	Depression (PHQ-2)	Anxiety (GAD-2)	Mental Distress (PHQ-4)
CFQ Total	0.261	0.486	0.447	0.498
CFQ Physical	0.265	0.503	0.453	0.511
CFQ Mental	0.208	0.367	0.358	0.387
CFQ Item				
1	0.209	0.416	0.382	0.426
2	0.19	0.389	0.39	0.416
3	0.193	0.413	0.394	0.431
4	0.279	0.487	0.428	0.489
5	0.282	0.491	0.413	0.484
6	0.189	0.369	0.294	0.355
7	0.219	0.406	0.376	0.418
8	0.242	0.399	0.384	0.418
9	0.138	0.243	0.224	0.249
10	0.135	0.265	0.267	0.284
11	0.167	0.3	0.308	0.324

*Note. N* = 2476–2504, All *p* ≤ .001. Bivariate Pearson correlations uncorrected for multiple testing.

### 3.4. Multiple regression

Results of multiple linear regression analyses are presented in Table 5. Older age, identifying as a woman, lower equivalized income, being unemployed, being religiously affiliated, not living in a steady partnership, and living in a multi-person household were statistically significantly associated with higher global fatigue severity. The same pattern was found for physical fatigue severity. For the mental subscale, however, gender, religious affiliation, being in a steady partnership, and living in a multi-person household were not statistically significantly associated with fatigue severity. Unemployment emerged as the strongest predictor of fatigue severity in the models testing for global, physical, and mental fatigue. The examined predictors explained only 4% of the variance in global, respectively physical fatigue, and only 2% of the variance in mental fatigue.

**Table 5.** Multiple linear regression analysis of fatigue severity on sociodemographic and socioeconomic characteristics.

	CFQ total (*N* = 2347)			CFQ physical (*N* = 2362)			CFQ mental (*N* = 2358)		
	β	se	*p*	β	se	*p*	β	se	*p*
Age	0.15	0.02	**<0.001**	0.16	0.02	**<0.001**	0.12	0.02	**<0.001**
Gender (men)	−0.10	0.04	**0.018**	−0.11	0.04	**0.006**	−0.05	0.04	0.216
Migration background (yes)	−0.02	0.06	0.769	0.01	0.06	0.911	−0.08	0.06	0.227
High school graduate (yes)	0.06	0.05	0.185	0.07	0.05	0.122	0.03	0.05	0.569
Equivalized household income	−0.08	0.02	**0.001**	−0.07	0.02	**0.002**	−0.07	0.02	**0.002**
Unemployment (yes)	0.38	0.09	**<.001**	0.36	0.09	**<0.001**	0.30	0.09	**<0.001**
Religiously affiliated (yes)	0.09	0.04	**0.048**	0.09	0.04	**0.044**	0.06	0.04	0.186
Living in metropolitan area (yes)	−0.01	0.05	0.818	−0.01	0.05	0.780	−0.01	0.05	0.917
Living in a steady partnership (yes)	−0.15	0.06	**0.016**	−0.17	0.06	**0.004**	−0.06	0.06	0.303
Living with children (yes)	−0.01	0.06	0.921	−0.01	0.06	0.904	−0.01	0.06	0.904
Living alone (yes)	−0.13	0.06	**0.045**	−0.13	0.06	**0.049**	−0.10	0.07	0.118
	*F* = 9.28, *df* (11, 2335), *p* < 0.0001, Adj. *R^2^* = 0.04			*F* = 9.73, *df* (11, 2350), *p* < 0.0001, Adj. *R^2^* = 0.04			*F* = 5.59, *df* (11, 2346), *p* < 0.0001, Adj. *R^2^* = 0.02		

*Note.* Statistically significant (*p* ≤ 0.05) associations are printed in bold. *β*: standardized beta coefficient, df: degrees of freedom, F: F-values of model’s F-statistic, se: standard error.

### 3.5. Fatigue case definition

Severe fatigue was reported by 14.2% (*N* = 354) of the sample. The six-month criterion for chronic fatigue was reported by 4.3% (*N* = 103) of the sample. Thus, severe fatigue without meeting the criteria for chronicity was reported by 10% (*N* = 251) of participants.

### 3.6. Norm values

Norm values are presented as percentile ranks stratified by age group and gender for total fatigue severity (Table S1) and the physical and mental subdimensions (Table S2).

## 4. Discussion

### 4.1. Chronic fatigue and psychometric properties of the CFQ

The present study investigated the psychometric properties of the German version of the CFQ in the general population. In summary, the CFQ has very good psychometric properties and is an efficient screening instrument for assessing the severity and chronicity of fatigue, which is of practical use in general practice but also in large-scale surveys. Several aspects are discussed below.

The slightly lower internal consistency of the mental subscale compared to the total and physical scale may be affected due to several reasons. First, it comprises a smaller number of items as compared to the physical and total scales, which is known to affect reliability [36]. Second, the anchors of item 11, as part of the mental subscale, have a different wording. It has the lowest part-whole correlation with the total scale and the lowest intercorrelation with the other items. Wording and item anchors may affect the internal consistency of instruments [37].

We replicated the proposed factor structure of the CFQ with a physical and a mental subdimension of fatigue. In line with conceptual considerations, this allows for a distinction between somatic aspects of fatigue, such as decreased energy and physical exhaustion, and mental or cognitive aspects, such as memory difficulties and speech problems. However, we also found support for the more parsimonious one-factor model. More generally, the dimensionality of fatigue has been debated, with some arguing for a further differentiation of emotional or motivational factors [38]. While the two-factor solution of the CFQ has been supported in previous studies [13,20,39], two Chinese studies reported that a three-factor solution distinguishing between general fatigue, specific fatigue aspects, and language difficulties [40] or physical fatigue, low energy, and mental fatigue [17] is superior. On the other hand, studies of the Multidimensional Fatigue Inventory (MFI-20), another popular fatigue measure, have argued for combining its inherent general (e.g. ‘I feel fit’) and physical fatigue (e.g. ‘Physically I feel only able to do a little’) subdimensions, due to a lack of discrimination between the two [23,41]. This is further substantiated by qualitative research highlighting that participants had difficulty clearly assigning some CFQ items to either physical or mental aspects of fatigue [42]. For many applications, a detailed examination of fatigue subdomains is necessary, but for certain clinical or research questions, a holistic assessment of fatigue may be more appropriate. Adding to previous findings, our results justify summing up the CFQ in a total severity score, while also examining its subfacets [43].

The physical subscale of the CFQ correlates more strongly with depression and anxiety scales, which is to be expected given the overlap of with the physical symptoms of depression. For the mental subdimension, the strongest intercorrelation was found for difficulty concentrating, which is also a symptom of many mental disorders, such as major depression and generalized anxiety disorder [44]. Overall, the strength of the associations between the CFQ and the PHQ-4 scales indicates a significant overlap, but also sufficient unique variation between the constructs. The relative independence of fatigue, depression and anxiety has also been reported in reviews and meta-analyses [45,46]. Therefore Brown et al. [47] recommended that cognitive, emotional and physical aspects of depression should be assessed separately, especially when CFS is suspected. In our study socio-demographic and socio-economic variables explained about 4% of the variance in fatigue severity, which is slightly lower compared to common findings on depression and anxiety (about 6%–7%) [48,49] and much lower compared to findings on constructs such as quality of life with 14%–30% [50,51]. Across all CFQ scales, higher fatigue was statistically significantly associated with older age, lower household income and unemployment. More global and physical fatigue was also associated with female gender, religious affiliation, not living in a steady partnership, and living in a multi person household. Previous research on the relevance of sociodemographic variables for explaining fatigue severity has been inconclusive [1,2, 8,11]. Representative studies from the German population show small but systematic age [20,41] and gender effects [20,41,52]. Overall, this is consistent with our findings, yet, gender was not associated with mental aspects of fatigue in our study, contrasts previous findings on the CFQ specifically [20]. It seems noteworthy, that current unemployment emerged as the strongest predictor of fatigue severity, highlighting its substantial health and psychosocial burden. This is in line with previous research showing a negative relationship between social status and fatigue [1,53].

Compared to data collected in 2004 with a similar procedure [20], we found less CF, while severe levels of fatigue increased. 16 years later 4.3% of the German population reported CF compared to 6.3% in 2004. Severe fatigue without reaching the criteria for chronicity was reported by 10% of our sample, compared to only 7.4% in 2004. In conclusion, in the present study we found a greater divergence between participants reporting severe and chronic fatigue levels. The finding may be related to the fact that our data were collected during the ongoing pandemic, between December 2020 and January 2021, when vaccination was not readily available and contact restrictions were implemented during the German holidays.

### 4.2. Strength and limitations

The representativeness and large sample size are major strengths of the present study. The findings are limited by the lack of a thorough examination of the validity of the instrument. Our criteria for discriminant validity do not include measures of somatic symptoms, such as pain, and general physical health. In addition, the measures included to assess anxiety and depression are brief screening instruments. The study is further limited by the lack of a gold standard to validate the presence of severe and chronic fatigue levels. Normative data could only be reported for individuals for men and women, as only four participants identified as gender diverse. Various medical conditions such as cancer, multiple sclerosis or mental health problems, may affect fatigue reporting. We did not exclude participants on the basis of reporting such a condition. This extends to infections with COVID-19, with the pandemic still ongoing at the time of data collection.

### 4.3. Conclusions

The CFQ has good psychometric properties in the general population. Our results support the use of a total score as well as the examination of physical and mental fatigue domains. The use of screening tools is an efficient way to identify cases of fatigue in clinical settings and to monitor fatigue in epidemiologic studies. Future studies should systematically compare fatigue levels across chronic health conditions.

## Supplementary Material

Supplement.docx

## Data Availability

Data is available upon reasonable request from the corresponding author.
