# Supplementary material for: Chronic fatigue: psychometric properties and updated norm values of the Chalder fatigue scale in a cross-sectional sample representative of the German population
Source: Ann Med. 2025 Jul 11;57(1):2524087. doi: 10.1080/07853890.2025.2524087 (PMC12258208; doi:10.1080/07853890.2025.2524087)
Supplement: Supplement.docx [file IANN_A_2524087_SM0209.docx]

**Supplement**

**Table S1 Normative data for the German general population, for the CFQ Total score**

| Sum Score | Percentile Rank | | | | | | | | | | | | | | |
| --- | --- | --- | --- | --- | --- | --- | --- | --- | --- | --- | --- | --- | --- | --- | --- |
|  | Total | Men | | | | | | | Women | | | | | | |
|  | 16-96 years | 16 -24 years | 25-34 years | 35-44 years | 45-54 years | 55-64 years | 65-74 years | ≥75 years | 16 -24  years | 25-34 years | 35-44 years | 45-54 years | 55-64 years | 65-74  years | ≥75 years |
|  |  |  |  |  |  |  |  |  |  |  |  |  |  |  |  |
|  | N = 2486 | n = 101 | n = 188 | n = 170 | n = 192 | n = 243 | n = 188 | n = 95 | n = 122 | n = 172 | n = 224 | n = 214 | n = 241 | n = 199 | n = 137 |
| 0 | 1.3 | 4.5 | 1.9 | 0.9 | 1.3 | 1.2 | 0.3 | 0 | 0.8 | 1.2 | 1.6 | 1.6 | 0.8 | 1.5 | 0.7 |
| 1 | 4.8 | 11.4 | 7.4 | 3.8 | 5.2 | 4.5 | 3.7 | 0 | 4.1 | 5.2 | 6 | 6.1 | 2.9 | 4.8 | 2.2 |
| 2 | 7.9 | 13.9 | 12.2 | 6.8 | 8.9 | 7.6 | 7.2 | 0 | 7.8 | 9 | 9.8 | 9.6 | 5 | 7.3 | 3.3 |
| 3 | 9.5 | 14.9 | 14.1 | 9.4 | 10.7 | 9.5 | 8 | 0.5 | 9.4 | 11.3 | 12.1 | 10.5 | 6.4 | 8.3 | 4.4 |
| 4 | 11.3 | 16.8 | 16 | 12.9 | 12 | 11.1 | 9.6 | 1.6 | 11.5 | 14.2 | 14.7 | 11.7 | 7.9 | 10.3 | 5.5 |
| 5 | 13.5 | 18.8 | 18.6 | 15.6 | 13 | 12.8 | 11.4 | 3.2 | 15.2 | 16.3 | 17.2 | 13.3 | 10.8 | 13.6 | 5.8 |
| 6 | 15.6 | 21.8 | 21.3 | 17.9 | 15.1 | 14.6 | 13.8 | 4.2 | 19.3 | 17.4 | 19.2 | 15.7 | 13.1 | 15.6 | 5.8 |
| 7 | 17.9 | 27.7 | 23.4 | 20.9 | 18 | 17.5 | 16.8 | 4.2 | 22.1 | 19.8 | 20.8 | 18.9 | 14.5 | 16.8 | 6.2 |
| 8 | 20.7 | 33.7 | 26.1 | 24.4 | 21.6 | 20.2 | 19.1 | 5.8 | 25 | 23.3 | 21.9 | 22.7 | 16.6 | 18.8 | 8.4 |
| 9 | 23.5 | 35.6 | 29.3 | 29.1 | 25 | 23.7 | 21.3 | 7.4 | 29.9 | 25.3 | 24.1 | 25 | 18.7 | 21.9 | 11.3 |
| 10 | 27.2 | 39.1 | 34 | 35 | 29.2 | 28.2 | 24.5 | 7.9 | 34.4 | 27.9 | 28.3 | 26.4 | 22.8 | 24.6 | 15 |
| 11 | 52.5 | 61.9 | 61.2 | 60.6 | 55.5 | 54.3 | 50.3 | 33.7 | 55.3 | 52.3 | 54 | 51.4 | 47.9 | 50 | 41.6 |
| 12 | 77.9 | 83.7 | 87.5 | 85.3 | 80.2 | 80.7 | 77.1 | 61.1 | 76.6 | 77 | 78.6 | 78.3 | 72.8 | 75.6 | 68.2 |
| 13 | 82 | 88.1 | 91 | 88.5 | 83.9 | 84.2 | 82.7 | 68.4 | 81.1 | 80.5 | 81.5 | 83.6 | 77.6 | 78.6 | 73.4 |
| 14 | 85.3 | 91.1 | 92.3 | 90.9 | 87.5 | 86.6 | 86.4 | 75.3 | 84.4 | 84 | 84.2 | 87.4 | 81.7 | 81.9 | 77 |
| 15 | 88 | 93.1 | 93.9 | 93.5 | 90.6 | 88.3 | 88.6 | 79.5 | 86.1 | 88.4 | 86.8 | 89.7 | 85.7 | 84.2 | 80.7 |
| 16 | 90 | 94.1 | 95.2 | 95 | 93 | 89.5 | 91 | 82.1 | 88.9 | 90.7 | 89.1 | 91.6 | 88 | 85.9 | 83.9 |
| 17 | 91.7 | 95 | 97.1 | 95.3 | 94.5 | 90.3 | 93.4 | 82.6 | 92.6 | 92.4 | 90.6 | 93.5 | 89.2 | 87.9 | 86.5 |
| 18 | 93.5 | 96.5 | 98.9 | 96.2 | 95.8 | 91.8 | 95.2 | 86.3 | 95.1 | 93.9 | 92 | 95.3 | 91.5 | 90.7 | 88.7 |
| 19 | 95 | 97 | 98.9 | 97.4 | 96.6 | 93.6 | 96.5 | 91.1 | 96.7 | 95.3 | 93.1 | 96.3 | 93.6 | 93.5 | 90.1 |
| 20 | 96 | 97.5 | 99.2 | 97.9 | 97.4 | 95.1 | 97.1 | 94.2 | 97.5 | 96.8 | 94.2 | 96.3 | 94.6 | 95.5 | 91.6 |
| 21 | 96.8 | 98 | 99.5 | 98.2 | 98.2 | 95.9 | 97.9 | 96.3 | 97.5 | 97.4 | 95.1 | 97 | 95.2 | 97 | 92.7 |
| 22 | 97.6 | 98 | 99.7 | 98.5 | 98.4 | 96.5 | 99.2 | 97.4 | 97.5 | 98.5 | 96.4 | 98.4 | 96.3 | 97.7 | 94.2 |
| 23 | 98.3 | 98 | 100 | 98.8 | 99 | 97.1 | 100 | 97.9 | 97.5 | 99.4 | 97.3 | 99.1 | 97.3 | 98.2 | 96.7 |
| 24 | 98.6 | 98 | 100 | 98.8 | 99.5 | 97.1 | 100 | 97.9 | 97.5 | 99.4 | 97.5 | 99.1 | 97.9 | 98.7 | 98.9 |
| 25 | 98.8 | 98.5 | 100 | 98.8 | 99.5 | 97.5 | 100 | 97.9 | 97.5 | 99.4 | 98 | 99.1 | 98.3 | 99 | 99.3 |
| 26 | 99 | 99 | 100 | 98.8 | 99.7 | 98.1 | 100 | 97.9 | 97.5 | 99.4 | 98.4 | 99.3 | 98.3 | 99.5 | 99.3 |
| 27 | 99.2 | 99 | 100 | 98.8 | 100 | 98.6 | 100 | 98.4 | 98 | 99.4 | 98.7 | 99.8 | 98.5 | 100 | 99.6 |
| 28 | 99.4 | 99 | 100 | 98.8 | 100 | 98.8 | 100 | 99.5 | 98.8 | 99.4 | 98.9 | 100 | 98.8 | 100 | 100 |
| 29 | 99.6 | 99.5 | 100 | 98.8 | 100 | 99 | 100 | 100 | 99.6 | 99.7 | 99.1 | 100 | 99 | 100 | 100 |
| 30 | 99.7 | 100 | 100 | 98.8 | 100 | 99.4 | 100 | 100 | 100 | 100 | 99.1 | 100 | 99.4 | 100 | 100 |
| 31 | 99.8 | 100 | 100 | 98.8 | 100 | 99.6 | 100 | 100 | 100 | 100 | 99.3 | 100 | 99.8 | 100 | 100 |
| 32 | 99.9 | 100 | 100 | 99.1 | 100 | 99.6 | 100 | 100 | 100 | 100 | 99.6 | 100 | 100 | 100 | 100 |
| 33 | 99.9 | 100 | 100 | 99.7 | 100 | 99.8 | 100 | 100 | 100 | 100 | 99.8 | 100 | 100 | 100 | 100 |

**Table S2 Normative data for the German general population, for the CFQ subscales (CFQ-physical, CFQ mental)**

|  | Percentile Rank | | | | | | | | | | | | | | |
| --- | --- | --- | --- | --- | --- | --- | --- | --- | --- | --- | --- | --- | --- | --- | --- |
|  | Total | Men | | | | | | | Women | | | | | | |
|  | 16-96 years | 16 -24 years | 25-34 years | 35-44 years | 45-54 years | 55-64 years | 65-74 years | ≥75 years | 16 -24  years | 25-34 years | 35-44 years | 45-54 years | 55-64 years | 65-74  years | ≥75 years |
|  |  |  |  |  |  |  |  |  |  |  |  |  |  |  |  |
|  | N = 2502 | n = 102 | n = 188 | n = 172 | n = 192 | n = 243 | n = 190 | n = 97 | n = 125 | n = 174 | n = 224 | n = 216 | n = 241 | n = 200 | n = 138 |
|  |  |  |  |  |  |  |  |  |  |  |  |  |  |  |  |
| 0 | 3.7 | 7.4 | 5.3 | 2.9 | 3.9 | 3.5 | 3.4 | 0 | 3.2 | 4.6 | 4.9 | 4.2 | 2.1 | 3.8 | 1.4 |
| 1 | 8.2 | 14.7 | 11.7 | 7.3 | 8.6 | 7.6 | 7.6 | 0 | 8 | 10.6 | 11.2 | 9.5 | 4.8 | 7.8 | 4 |
| 2 | 10.3 | 15.2 | 14.1 | 11 | 10.9 | 9.3 | 9.5 | 0 | 11.6 | 13.5 | 13.8 | 11.3 | 6.8 | 8.8 | 5.4 |
| 3 | 12.8 | 19.1 | 18.1 | 15.1 | 13.8 | 11.3 | 11.6 | 1 | 15.6 | 15.2 | 16.5 | 13.4 | 9.1 | 11 | 5.8 |
| 4 | 16 | 26 | 22.3 | 19.8 | 16.9 | 14.8 | 14.2 | 3.6 | 20.8 | 17.5 | 18.8 | 16.9 | 11.8 | 14 | 5.8 |
| 5 | 19.8 | 32.8 | 25.5 | 25.3 | 21.6 | 18.5 | 18.2 | 5.2 | 26.4 | 21.6 | 21 | 20.6 | 14.7 | 18 | 7.6 |
| 6 | 24.3 | 39.2 | 30.1 | 31.4 | 27.9 | 23 | 22.9 | 6.2 | 30.8 | 26.1 | 25.2 | 23.8 | 18.5 | 22.2 | 12 |
| 7 | 51.7 | 62.7 | 59 | 59.6 | 55.7 | 52.5 | 51.1 | 34.5 | 52.4 | 50.6 | 52.9 | 50.5 | 46.7 | 50 | 40.9 |
| 8 | 78.9 | 86.3 | 88 | 86 | 82 | 80.9 | 79.5 | 64.4 | 74.8 | 75.6 | 79.5 | 78.7 | 75.3 | 77.2 | 69.9 |
| 9 | 83.5 | 90.2 | 92.6 | 89 | 85.9 | 85.6 | 84.2 | 71.1 | 80.4 | 80.5 | 83.3 | 84.7 | 80.3 | 80.5 | 74.6 |
| 10 | 86.8 | 92.2 | 94.9 | 91.6 | 89.6 | 88.3 | 88.2 | 77.8 | 85.2 | 84.8 | 85.7 | 88.4 | 84 | 83 | 77.5 |
| 11 | 89.2 | 94.1 | 96.3 | 93.9 | 92.2 | 89.5 | 91.1 | 80.9 | 88.4 | 88.8 | 87.7 | 90.3 | 86.9 | 85 | 80.8 |
| 12 | 91.5 | 96.1 | 97.3 | 95.1 | 93.8 | 91.4 | 93.4 | 83 | 91.2 | 91.1 | 90.8 | 92.8 | 89.8 | 87.8 | 85.1 |
| 13 | 93.7 | 97.5 | 97.9 | 96.2 | 95.1 | 93 | 95.8 | 88.1 | 94 | 92.8 | 93.3 | 94.9 | 92.3 | 91 | 88.4 |
| 14 | 95.7 | 98 | 98.4 | 97.4 | 96.9 | 94.9 | 97.6 | 92.8 | 95.6 | 95.1 | 95.5 | 95.8 | 94.2 | 95.2 | 92 |
| 15 | 97.3 | 98 | 99.5 | 98 | 98.4 | 96.1 | 98.7 | 94.8 | 96.4 | 97.4 | 97.1 | 97.5 | 95.6 | 98 | 95.3 |
| 16 | 98 | 98 | 100 | 98.5 | 99 | 96.9 | 99.2 | 96.9 | 96.8 | 98.3 | 97.5 | 98.8 | 96.5 | 98.5 | 96.7 |
| 17 | 98.6 | 98 | 100 | 98.8 | 99.5 | 97.9 | 99.5 | 97.9 | 97.2 | 98.9 | 98 | 99.3 | 97.7 | 99.2 | 97.8 |
| 18 | 99.1 | 99 | 100 | 98.8 | 100 | 98.4 | 99.7 | 97.9 | 98 | 99.4 | 98.2 | 99.8 | 98.3 | 100 | 99.3 |
| 19 | 99.3 | 100 | 100 | 98.8 | 100 | 98.6 | 100 | 98.5 | 98.8 | 99.7 | 98.2 | 100 | 98.5 | 100 | 100 |
| 20 | 99.5 | 100 | 100 | 98.8 | 100 | 98.8 | 100 | 99 | 99.6 | 100 | 98.7 | 100 | 99 | 100 | 100 |
| 21 | 99.8 | 100 | 100 | 99.4 | 100 | 99.4 | 100 | 99.5 | 100 | 100 | 99.6 | 100 | 99.6 | 100 | 100 |
|  |  |  |  |  |  |  |  |  |  |  |  |  |  |  |  |
| CFQ Mental |  |  |  |  |  |  |  |  |  |  |  |  |  |  |  |
|  | N=2498 | n=101 | n=189 | n=172 | n=195 | n=244 | n=188 | n=96 | n=122 | n=172 | n=225 | n=214 | n=243 | n=199 | n=138 |
| 0 | 1.9 | 5.9 | 4 | 1.5 | 1.8 | 1.8 | 0.3 | 0.5 | 2 | 1.5 | 2 | 2.1 | 1.4 | 2 | 0.7 |
| 1 | 9.1 | 15.8 | 13.2 | 9 | 8.2 | 9.6 | 6.1 | 3.6 | 10.2 | 9.6 | 9.8 | 10.3 | 8.2 | 9.5 | 4 |
| 2 | 17.4 | 23.8 | 22.2 | 18.3 | 16.4 | 18 | 14.1 | 6.8 | 20.5 | 20.6 | 18.9 | 18.7 | 15.8 | 16.8 | 8.7 |
| 3 | 22.8 | 30.2 | 28.8 | 25.3 | 22.6 | 24.4 | 18.9 | 8.3 | 28.3 | 27.6 | 24.4 | 23.6 | 20.2 | 20.6 | 12.7 |
| 4 | 55.9 | 59.4 | 61.4 | 60.8 | 56.2 | 58 | 53.5 | 43.2 | 58.2 | 58.7 | 56 | 58.4 | 52.5 | 54 | 47.1 |
| 5 | 89.6 | 90.1 | 93.7 | 94.8 | 90.3 | 90 | 89.9 | 81.8 | 88.9 | 90.7 | 88 | 92.3 | 86.6 | 88.9 | 84.1 |
| 6 | 94.2 | 95 | 96.8 | 97.7 | 95.1 | 93.2 | 95.5 | 90.1 | 94.7 | 95.6 | 92 | 95.1 | 92.4 | 93.7 | 90.9 |
| 7 | 96.4 | 97.5 | 97.6 | 98.5 | 97.7 | 95.3 | 97.6 | 95.3 | 96.7 | 98 | 94.2 | 97.4 | 95.1 | 95.5 | 94.6 |
| 8 | 98 | 99 | 98.7 | 98.8 | 99 | 96.7 | 98.9 | 98.4 | 98 | 99.1 | 96.7 | 98.8 | 97.3 | 96.7 | 97.5 |
| 9 | 99.1 | 99 | 99.7 | 98.8 | 99.7 | 97.7 | 99.7 | 100 | 98.4 | 99.4 | 98.7 | 99.5 | 99 | 98.7 | 99.3 |
| 10 | 99.6 | 99 | 100 | 98.8 | 100 | 98.8 | 100 | 100 | 99.2 | 99.7 | 99.3 | 100 | 99.4 | 100 | 99.6 |
| 11 | 99.8 | 99.5 | 100 | 99.1 | 100 | 99.4 | 100 | 100 | 100 | 100 | 99.6 | 100 | 99.8 | 100 | 100 |
| 12 | 99.9 | 100 | 100 | 99.7 | 100 | 99.8 | 100 | 100 | 100 | 100 | 99.8 | 100 | 100 | 100 | 100 |
